# Nonviral Locally Injected Magnetic Vectors for In Vivo Gene Delivery: A Review of Studies on Magnetofection

**DOI:** 10.3390/nano11051078

**Published:** 2021-04-22

**Authors:** Artem A. Sizikov, Marianna V. Kharlamova, Maxim P. Nikitin, Petr I. Nikitin, Eugene L. Kolychev

**Affiliations:** 1 Moscow Institute of Physics and Technology, 141701 Dolgoprudny, Russia; aasizikov88@gmail.com (A.A.S.); mv.kharlamova@gmail.com (M.V.K.); max.nikitin@phystech.edu (M.P.N.); 2 Sirius University of Science and Technology, 354340 Sochi, Russia; 3 Prokhorov General Physics Institute of the Russian Academy of Sciences, 117942 Moscow, Russia

**Keywords:** magnetofection, magnetic nanoparticles, gene delivery

## Abstract

Magnetic nanoparticles have been widely used in nanobiomedicine for diagnostics and the treatment of diseases, and as carriers for various drugs. The unique magnetic properties of “magnetic” drugs allow their delivery in a targeted tumor or tissue upon application of a magnetic field. The approach of combining magnetic drug targeting and gene delivery is called magnetofection, and it is very promising. This method is simple and efficient for the delivery of genetic material to cells using magnetic nanoparticles controlled by an external magnetic field. However, magnetofection in vivo has been studied insufficiently both for local and systemic routes of magnetic vector injection, and the relevant data available in the literature are often merely descriptive and contradictory. In this review, we collected and systematized the data on the efficiency of the local injections of magnetic nanoparticles that carry genetic information upon application of external magnetic fields. We also investigated the efficiency of magnetofection in vivo, depending on the structure and coverage of magnetic vectors. The perspectives of the development of the method were also considered.

## 1. Introduction

Magnetic nanoparticles (MNPs) have been extensively used for various in vitro applications, and their unique abilities to respond to magnetic fields make them especially attractive for in vivo theranostics [1,2,3,4,5,6]. In vivo magnetofection, i.e., the delivery of genetic material with magnetic particles controlled by an external magnetic field, is a promising approach to significantly boost the efficiency of gene therapy. In gene therapy, a disease or cellular neoplasm can be treated by delivering the genetic material to specific cells in order, for instance, to increase the expression of specific genes or to reduce the production of a desired protein [7,8]. It is obvious that the direct delivery of nucleic acids to the targeted cells is the limiting factor of such a therapy [9,10,11]; therefore, the choice of a reliable and effective viral or nonviral vector [12,13,14] for delivery is very important. Commonly used viral vectors based on adenoviruses, lentiviruses, and adeno-associated viruses [15,16,17,18] are excellent carriers, but they suffer from a number of serious disadvantages, such as immunogenicity and carcinogenicity [19,20]. Nonviral vectors are safer; they include polyplexes based on cationic or neutral biodegradable polymers, lipoplexes (cationic liposomes and niosomes), complexes of deoxyribonucleic acid (DNA) with dendrimers or peptides by themselves [21,22], as well as combinations of all the above-mentioned vectors with magnetic particles [23,24]. In this case, magnetic particles themselves can be used for tumor therapy, for the targeted drug delivery to a selected part of the body, or for the magnetic separation of cells [25,26,27,28]. 

**Figure 1:**
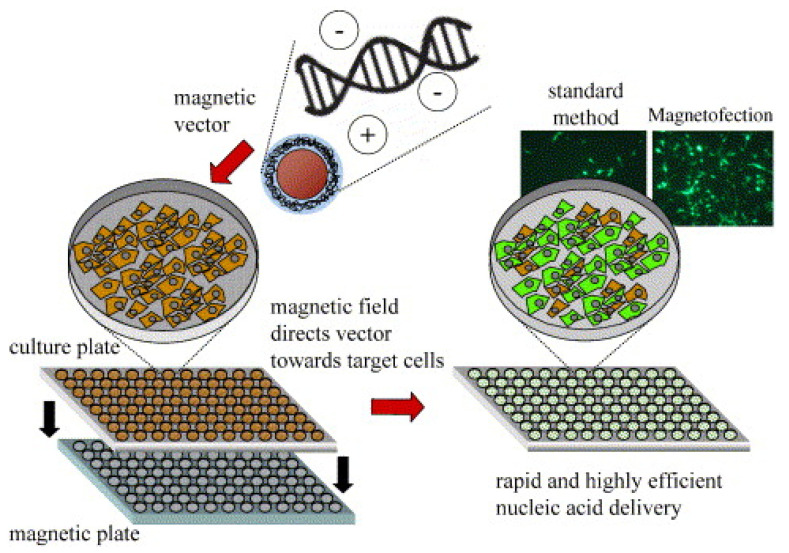
Principle of magnetofection in cell culture. Polyelectrolyte-coated magnetic nanoparticles are mixed with naked nucleic acids or synthetic or viral nucleic acid vectors in salt containing a buffer. The particles associate with the nucleic acids and vectors by electrostatic interaction and/or salt-induced colloid aggregation. The mixtures are added to cells in culture. The cell culture plate is positioned on a magnetic plate during 5–30 min of incubation. The magnetic plate consists of 96 individual neodymium–iron–boron magnets inserted in drill holes in an acrylic glass or PVC (polyvinyl chloride) plate in a strictly alternating polarization. The magnetic field rapidly sediments vectors on the cells to be transfected/transduced. The result is rapid kinetics and high-efficiency nucleic acid delivery. Adapted with permission from [52]. Copyright Elsevier, 2005.

As stated above, complexes based on magnetic particles and nucleic acids have multimodal properties; therefore, in addition to the direct gene delivery using such complexes, it is possible to carry out the magnetically controlled accumulation and release of particles [29,30,31], particle tracking by magnetic resonance imaging (MRI) [32,33,34,35,36], the imaging of tumors [37,38], and magnetic particle quantification (MPQ) studies [32,39,40,41], as well as magnetofection [9,42,43]. The term “magnetofection” refers to the use of a magnetic field and magnetic particles to improve the efficiency of gene delivery [44,45,46,47]. The principle of the method is clear: the magnetic field promotes the accumulation and retention of magnetic particles in the area of its application (a schematic conception of magnetofection is shown in Figure 1). Compared to conventional transfection based on polymers or lipids, magnetofection benefits from a number of obvious advantages, such as a higher efficiency and, consequently, a lower required dose of nucleic acid, shorter delivery time, and the ability to transfect locally and in a limited area [9,48,49,50]. As for the comparison of advantages and disadvantages of MNPs among other inorganic nanoparticles (gold nanoparticles, silica nanoparticles, quantum dots, etc.) for in vivo gene delivery, there are several comprehensive reviews with detailed tables [8,26,51].

All these properties are especially relevant when carrying out in vivo magnetofection. There are a number of reviews in the literature that are dedicated to various aspects of the nonviral delivery of genetic information, such as the properties and characteristics of vectors for in vitro magnetofection [9,47,52,53,54,55,56,57], the mechanism and distinctive features of transfection (magnetofection) [9,26,47,49,51,52,56,57,58,59,60,61,62,63], in vivo [8,26,51,58,59,61,64,65,66,67,68,69,70,71,72] and in vitro [8,26,51,61,64,65,67,69,71,72,73,74,75,76,77,78] transfection based on polyplexes and lipoplexes, and transfection with peptides [8,67,79]. There are no reviews on in vivo magnetofection. 

The main aim of this paper was to review the articles describing the techniques of in vivo magnetofection (transfection using a magnetic field). We should primarily note some boundary conditions. This paper reviews only those articles in which: (1) injection directly into a targeted tumor or tissue was used (the reasons we have only considered the local injection and did not touch the systemic injection are discussed shortly in the Conclusions and Perspectives section); (2) the authors directly used a magnetic field to deliver particles together with nucleic acid molecules; (3) the results of the experiments obtained with and without a magnetic field were compared. It is worth noting that the type of nucleic acid is not important for us, as it is known that, for magnetofection (at least for in vitro magnetofection), one can use plasmid DNA (pRNA), small interfering RNA (siRNA), short hairpin RNA (shRNA), and antisense oligonucleotides [80,81,82]. It is also not important for us what happens to the nucleic acid after its delivery to the cell wall of the target cell (reviews on the mechanisms of transfection are cited above). It is only important what (in addition to the magnetic core) the magnetic vector is built from, how efficiently the carrier of genetic information is delivered, and how the magnetic field affects this process. 

For the convenience of the reader, the reported results are summarized in a table supported by explanatory text. This promotes a better understanding of the idea of the current state of research in the field of in vivo magnetofection, existing challenges, as well as perspectives of the method development.

## 2. Applications of Magnetofection In Vivo

The aim of the review was to collect and analyze data on the promising method of nonviral gene delivery—magnetofection. The method is relatively “young,” and research on this method is still at the preclinical stage. We have managed to find only a few works that demonstrate the value and efficiency of this method for the delivery of genes to tissues and tumor models in vivo, and authors have rarely indicated the strength and spatial distributions of the magnetic field used, which are important parameters, as only the gradient of the magnetic field creates a force acting on the MNP. All selected articles are collected in Table 1 (we searched the words “magnetofection in vivo,” “magnetic delivery in vivo,” “magnetic gene delivery in vivo,” and “magnetic tumor targeting in vivo” in Web of Science).

For readability, we divided the results into three groups: magnetopolyplexes (a vector comprising magnetic nanoparticles coated with polymers), magnetoliposomes (a vector comprising magnetic nanoparticles coated with lipids), and “unusual” examples of magnetic carriers (an unconventional magnetic core of the magnetocomplex, a complex possessing one more way of active targeting, etc.).

### 2.1. Magnetopolyplexes

**Figure 2:**
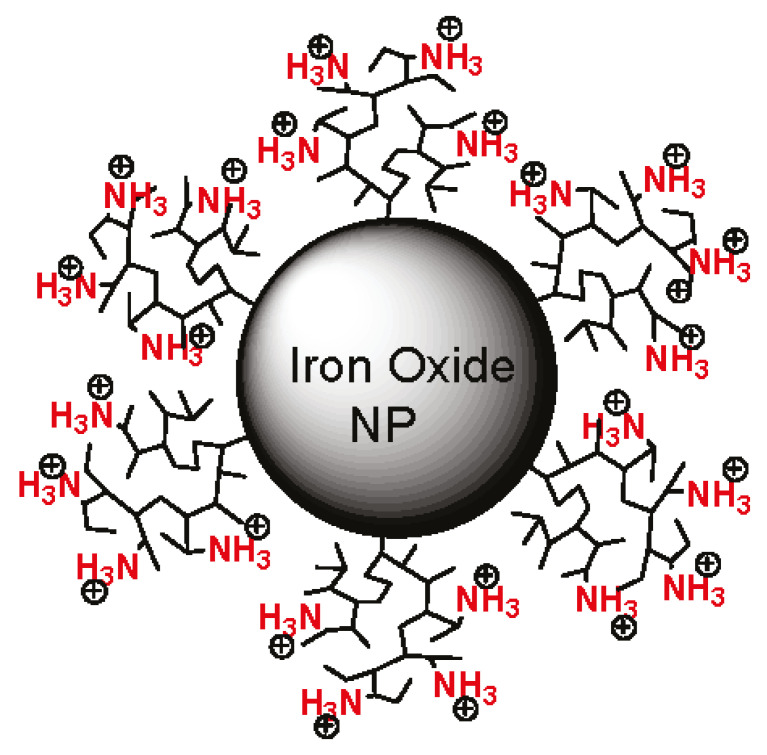
An example of magnetopolyplexes. Magnetic iron oxide nanoparticle coated with polyethylenimine. Adapted with permission from [112]. Copyright American Chemical Society, 2011.

The bulk of the work is dedicated to the study of magnetofection in vivo using magnetic particles (usually SPIONs) coated with cationic polymers [46,83,84,85,86,89,90,91,92,93,95,96,99,102,103] (Table 1). We start with an article on the method [46] where the author tried to apply the principles of magnetic drug targeting for gene delivery [105]. In Ref. [46], it was shown only at a qualitative level that X-gal staining performed 48 h after the gene delivery to the ilea of rats revealed efficient gene delivery only in the presence of a magnetic field. Additionally, it was found that magnetofection in the ear veins of pigs (as a model for gene delivery to endothelial cells) leads to a noticeable luciferase expression, while no luminescence was observed in the absence of a magnetic field. As for the magnetic vector itself, these were transMAG^PEI^ (Chemicell) particles—superparamagnetic iron oxide nanoparticles with an average size of 200 nm (by dynamic light scattering), coated with polyethylenimine (PEI, 800 kDa). PEI is the most effective nonviral vector due to its favorable characteristics of DNA protection, cell binding and uptake, and endosomal escape and release from the carrier [106,107,108]; an example of the structure of a PEI-coated magnetic particle is shown in Figure 2. Therefore, in most studies, magnetic particles coated with PEI are used. Using magnetopolyplexes consisting of the same transMAG^PEI^ (Chemicell) particles, the authors of Ref. [102] found that the level of luciferase expression in vivo was only slightly higher for magnetofection than for both naked DNA and magnetic particles without magnetic-field injection at 1, 7, and 14 days. The same nanoparticles were used by the authors of [92,93]. It was noted that magnetofection was used only as a tool to prevent the plasmid from spreading throughout the body. The aim of the work [92] was to attempt to determine the toxicity and feasibility of gene therapy with feline granulocyte-macrophage colony-stimulating factor (feGM-CSF) in cats with fibrosarcomas, as well as to establish a safe dose of a magnetic drug containing a plasmid, which is well tolerated and, thus, can be used safely in a subsequent second phase of the clinical trial [93]. Continuing the topic of magnetic vectors based on commercial particles (now from Oz Biosciences), NeuroMag magnetic nanoparticles (efficient in transfecting a large variety of primary neurons such as cortical, hippocampal, dorsal root ganglion, and motor neurons with all types of nucleic acids) and PolyMag (a cationic polymer-based magnetic nanoparticles formulation, designed for in vivo targeted transfection of nucleic acids) should be mentioned, which were used in Refs. [84,85], respectively. The authors of [84] state that neuronal transfection rates are comparable to those displayed by viral vectors [109,110,111]. This was the first study reporting the potential of magnetic nanoparticles to deliver a pDNA-containing channel rhodopsin gene into the CNS in a safe, efficient, and enduring manner.

Moving from commercial to custom-made particles, we start with a study [95] where a plasmid pACTERT-TRAIL was created, which used the human telomerase reverse transcriptase promoter, a tumor-specific promoter, to drive a tumor necrosis factor-related apoptosis-inducing ligand (TRAIL). The delivery itself was carried out using the Fe_3_O_4_-PEI-plasmid complex. PEI-modified iron oxide nanoparticles were prepared by alkaline co-precipitation using 25 kDa of branched PEI. As a result of studying the antitumor activity of hTRAIL in vivo, it was found that on the 32nd day after the injection of particles, the volume of the control tumor (PBS) was twice the volume of the tumor (Fe_3_O_4_-PEI-pACTERT-TRAIL + magnetic field); when using the same magnetic complex without the field, the tumor size was about the same as when using only the PBS, or 10–15% less. Similar to PEI, poly-(alkylacrylic acid) polymers, including noncytotoxicpolyacrylic acid (PAA), have been considered as endosomolytic polymers [113]. It has been shown that the inclusion of PAA to PEI-DNA transfection complexes not only increased the reporter gene expression, but also reduced toxicity in vivo [114]. Therefore, the GregorSersa group showed that surface-modified superparamagnetic iron oxide nanoparticles (SPIONs) with a combination of polyacrylic acid (PAA) and polyethylenimine (PEI) (SPIONs-PAA-PEI) proved to be safe and effective for the magnetofection of cells and tumors in mice [96]. The synthesis of SPION particles was carried out according to the Massart method [115] with subsequent coating with poly-(acrilyc acid) (8 kDa). The incubation of SPIONs-PAA with PEI (branched, 25 kDa) to obtain the SPIONs-PAA-PEI complex was carried out immediately before the magnetofection of tumors with pDNAIL-12, a plasmid that encodes immunostimulatory cytokine interleukin 12 (IL-12) to stimulate an immune response against a tumor. As a result of the application of the magnetic complex, the tumor growth delay on the 10th day after the last injection was: 0–1 days without a magnetic field and 7–9 days with a magnetic field. The same group led by Lara Prosen used the SPIONs-PAA-PEI complex to deliver plasmid DNA encoding short hairpin RNA against MCAM (the melanoma cell adhesion molecule) to explore the antitumor and antiangiogenic effects in vivo on melanoma tumors in mice [89], and to explore the distribution, accumulation, uptake, and the consequent therapeutic effect [90]. The tumor growth delay with the use of nanoparticles and a magnetic field for transfection after the last injection was: 3–5 days on day 10 [89] and 2–4 days on day 7 [90].

One of the problems faced by transfection researchers is severe particle aggregation, which restricts its further application in vivo. In this regard, the authors of Ref. [91] synthesized two types of magnetic particles and discussed what the degree of aggregation of magnetic nanoparticles (MNPs) may depend on. MNPs were synthesized by two different methods in aqueous [116] and organic media [117] with subsequent modification by ligand exchange or by silane-coupling agents to prepare the negatively charged coating, as this N-(trimethoxysilylpropyl) ethylenediaminetriacetic acid was used. As a result, when a complex of such particles with pCMV-Luc was introduced locally into the tumor, the luciferase activity 48 h after injection was 15 times higher in the case of magnetofection compared to transfection with the same complex without a field.

It is known that poly(lactic-co-glycolic acid) and polyethylenimine (PLGA-PEI)-coated magnetic nanoparticles as a nonviral gene vector can self-assemble DNA, and they are more stable, easier to manipulate, and more economic than cationic liposomes [118]. It is also known that the surface modification of PLGA-PEI/DNA composite nanoparticles through PEGylation (coating with poly-(ethylene glycol)) can reduce their cytotoxicity and enhance the systemic duration in vivo and plasmid DNA expression [119,120]. In this regard, the authors of Ref. [83] chose PEGylated MNP-PLGA-PEI magnetic nanoparticles as a transfection agent for magnetofection in primary hippocampal neurons. During the synthesis at the first stage, oleic acid-modified magnetic nanoparticles (Fe_3_O_4_) were synthesized following [121]; then, magnetic PLGA-PEI composite nanoparticles were prepared using the modified water-in-oil-in-water (w/o/w) double emulsion-solvent evaporation (DESE) method [122,123]. As a result, 10 days after the introduction of the complex magnetic vector with DNA, the fluorescent signal in the hippocampus of mice was noticeable only when using MNP-PLGA-PEI-PEG nanoparticles under the influence of a magnetic field. At the same time, perhaps due to the lower strength of the applied magnetic field [124], there was no fluorescence in mice treated with peptide-modified (such as neurotensin, VSV, TAT, or T7) magnetic nanoparticles, MNP-PLGA-PEI nanoparticles without using magnetic fields, or lipoplexes based on commercial Lipofectamine 2000/3000/iMAX kits. 

One of the properties that a polymer covering a magnetic core should possess is the ability to protect DNA from enzymatic degradation. In order to protect DNA in the Fe_3_O_4_ –PEI—pDNA complex, a poly-b-amino ester (PBAE) can be used, a hydrolytically biodegradable polymer, which has a high transfection efficacy specifically with adipose-derived stromal cells (ASCs) [125,126]. The authors of Ref. [86] did just that and chose MNP—PEI—PBAE (this is actually Fe_3_O_4_—PEI—pDNA coated with PBAE on top) as the basis for the magnetofection agent. To facilitate in situ transfection, nanoparticle-complexed Bcl-2/GFP minicircles were incorporated into a hydroxyapatite-coated poly-(lactic-coglycolic acid) (HA-PLGA) scaffold [127,128] onto which freshly harvested ASCs could be seeded. The present study investigated the effects of minicircle-mediated Bcl-2 up-regulation using magnetofection on osteogenic differentiation and using a novel prefabricated scaffold to transfect ASCs after implantation in a spatiotemporally controlled manner to promote bone regeneration. As a result, the use of PBAE coated particles in the presence of a magnetic field resulted in the highest transfection efficiency (30.6%) compared to the transfection efficiency without a field (˂5%), and a significant improvement in bone regeneration 8 weeks after surgery. Next, let us consider another study on bone defects [99], where chitosan (a linear polysaccharide containing amino groups) here was used as a cationic polymer covering the magnetic core. The SPIONs-chitosan complex was prepared by the co-precipitation method [129,130,131]. The very essence of the work was that a new artificial bone framework loaded with magnetic microspheres consisting of a magnetic nanocomplex and a plasmid was developed and confirmed here. Microspheres vibrated under the influence of a static and oscillating magnetic field, which promoted the release of plasmid genes from microspheres for transfection of the surrounding cells, which led to the expression of the vascular endothelial growth factor protein, thereby contributing to the improvement of angiogenesis and osteogenesis within the scaffold, internal vascularization of the artificial bone scaffold, and repair of large bone defects. The system worked better with the magnetic field than without, but it was difficult to quantify the results.

### 2.2. Magnetolipoplexes

**Figure 3:**
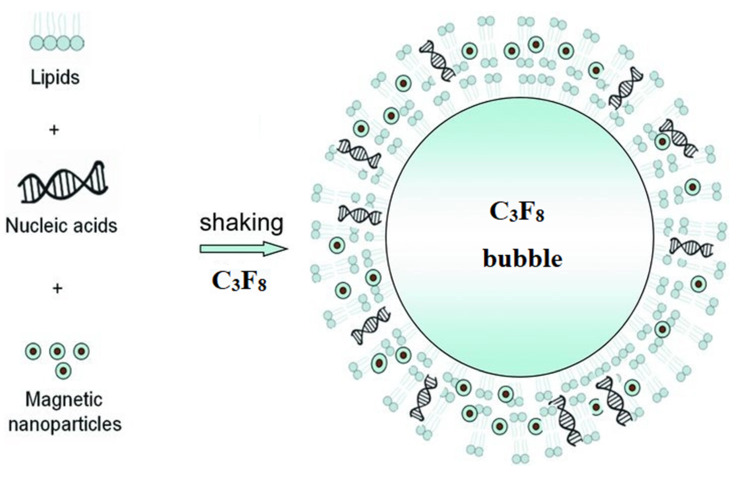
Schematic representation of the self-assembly of the lipids, magnetic nanoparticles, and nucleic acids into magnetic, acoustically active lipospheres upon shaking of the components in an aqueous medium in the presence of perfluoropropane. Adapted with permission from [133]. Copyright John Wiley and Sons, 2010.

The use of magnetolipoplexes turned out to be a much less popular method of the local delivery of information by magnetofection. Only a couple of examples were found in the open literature. In Ref. [97], cationic lipid 67 (GL67)-pDNA complexes or naked pDNA were coupled to transMAGPEI (Chemicell). Surprisingly, as a result of magnetofection in the nose of a mouse in vivo, according to the analysis of luciferase activity, the best result was shown by the GL67/pDNA lipoplex. Moreover, the addition of magnetic particles to the GL67/pDNA complexes led to a very significant 50-fold decrease in gene expression, while the expression did not increase even when using a magnetic field. A possible reason is the formation of relatively large aggregates of particles that settle at the injection site and cannot release DNA. In Ref. [100], perfluoropropane-filled magnetic lipospheres (“magnetobubbles”) from Tween60-coated magnetic nanoparticles, Metafectene, soybean-oil, and DNA were prepared (Figure 3). The authors studied the effect in an oversized random-pattern-flap model in rats. According to the results of the study, the authors stated that magnetofection of the VEGF165 gene using acoustically active magnetic lipospheres led to an increased VEGF protein concentration in the target tissue, induced an enhanced blood flow, and resulted in a reduced rate of necrosis in this setting. Moreover, the maximum result (comparable to the adenoviral vector [132]) was obtained only by the combination of a magnetic field and ultrasound.

### 2.3. “Unusual” Examples of Magnetic Carriers

This section contains examples of the use of unusual magnetic media or the addition of another type of active targeting (in addition to the magnetic field). We start with studies [87,88] in which the authors proposed a synthesis method and protocols for using, including in vivo, a combined magnetic vector—magnetic calcium phosphate (‘CaP’) nanoparticles (NPs). Calcium phosphate has been used for transfection for a long time [134,135]. It is biocompatible, biodegradable, and easy to obtain, but the transfection efficiency is relatively low [136], so the authors suggested that the use of another type of active targeting (magnetic field) would improve the results of transfection. Ferucarbotran, which is a carboxydextran-coated superparamagnetic maghemite (γ-Fe_2_O_3_) nanocrystal (Resovist, KYOWA CritiCare Co., Ltd., Kanagawa, Japan), was used to synthesize the combined magnetic vector. It is worth noting that ferucarbotran is a clinically approved MRI contrast agent of the liver; therefore, the resulting particles potentially have multimodal properties. As another source of magnetic calcium phosphate (‘CaP’) nanoparticles (NPs), supersaturated ‘CaP’ solutions were used [137]. As in most of the cases discussed above, the results of the work were determined using the luciferase assay. It has been shown reliably in vitro that the addition of a magnetic moiety to a transfection agent does improve results [87]. As for the in vivo results [88], there is a detailed protocol, a statement that everything works, but, unfortunately, there are no pictures or numbers in the article.

In Ref. [48], the gene transfer was successfully completed by magnetofection using self-assembled ternary complexes of cationic magnetic nanoparticles, plasmid DNA, and cell-penetrating bis(cysteinyl)histidine-rich, endosomolytic Tat peptides [138] (as another type of active targeting). To prepare magnetofection complexes solutions of plasmid DNA and PolyMag (Chemicell) (magnetic nanoparticles coated with PEI 25 kDa) were mixed followed by the addition of the endosomolytic Tat peptides. As a result, an increased transfection efficiency was observed in vivo in the spinal cord of rats after intrathecal injection into the lower back. The resulting magnetofection complexes, injected into the cerebrospinal fluid, reacted to a moving magnetic field, shifting from the injection site (determined by the expression of the transgene in an area distant from the injection) when the magnet moved along the spine. Under the influence of a magnetic field, the level of transgene expression by the PolyMag-Tat-pDNA complex was approximately 2 times higher than that of the binary complexes (without Tat), and also higher than those of the classical PEI-DNA and Lipofectamine2000-DNA. If the magnet is moved along the spine, the effectiveness of magnetofection doubles.

**Figure 4:**
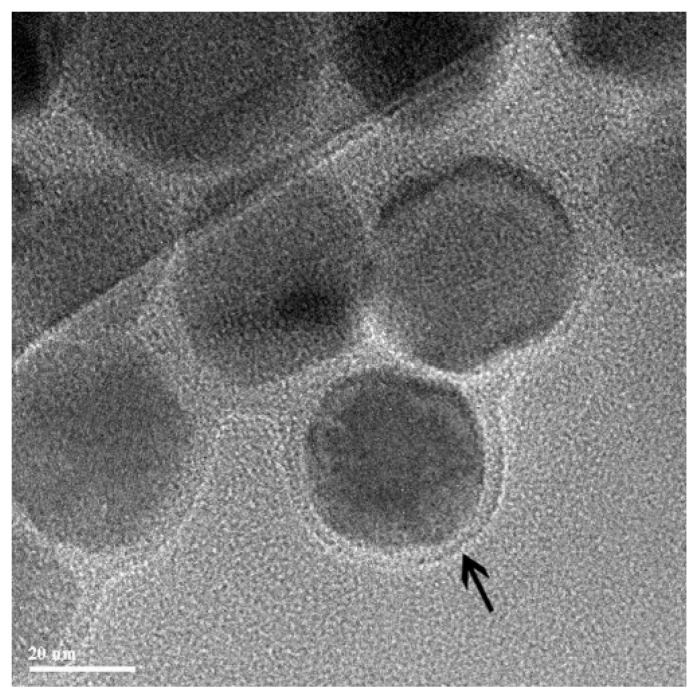
TEM image of bacterial magnetic particles enveloped by the cytoplasmic membrane (arrow). Adapted with permission from [101]. Copyright John Wiley and Sons, 2007.

The authors of [98] suggested magnetic gold nanoparticles (MGNs) as unusual and novel nonviral gene carriers. They used MGNs and the mediated plasmid pGPH1/GFP/Neo-Bag-1-homo-825 silencing Bag-1 gene for treating colorectal cancer in vivo and in vitro. It should be noted that the proposed magnetic complex for the delivery of plasmid DNA worked (determined by a significant slowdown in tumor growth compared to the control groups); however, the presence or absence of a magnetic field practically did not affect the quantitative result. A possible explanation for this result is the local introduction of the magnetochemical complex. The authors argue that this itself is a good way to avoid significant diffusion of the drug dose when injected into solid tumors. Another unusual “core” of the magnetic vector is bacterial magnetic particles (BMPs) [101]. BMPs as types of novel nanomaterials are made of Fe_3_O_4_ (Figure 4), and they are enveloped by the cytoplasmic membrane in Magnetospirillum gryphiswaldense MSR-1 [139]. Based on the experiments in vivo, it was shown that BMPs-PEI/DNA complexes plus a magnetic field could enhance the gene expression of the pCMVβ plasmid in leg muscles (the results are qualitative, but nothing can be said quantitatively) [101]. In a mouse tumor model [100], the subcutaneous injection of BMP-V (bacterial magnetic particles vaccine) plus magnetic exposure elicited systemic HPV-E7-specific immunity, leading to significant tumor growth inhibition. The polyplexes of DNA, PEI, and BMPs were prepared at mass ratios of DNA: PEI: BMP = ¼:1:0.3. The results show that the expression of a luciferase reporter was clearly observed in the tissue injected with BMPs-pGL4.17 with the magnet as compared with those without the magnet.

## 3. Conclusions and Perspectives

As it is noted in the introduction, the main aim of this mini-review was to collect and analyze data influencing magnetofection in vivo processes. In this regard, we selected articles on in vivo magnetofection and considered the structure of each magnetic vector, depending on the type of coating of the magnetic core. The efficiency of delivery of the carrier of genetic information and the influence of the magnetic field on this process were not clear. In vivo magnetofection, which is a nonviral, noninvasive, and painless system for the delivery of genetic information, is a very promising system. However, it was not clear why the authors who performed transfection, including in vivo transfection, did not use a magnetic field in their studies. This was mentioned in the review published in 2011 [9], where there were only two examples where a magnetic field was used among several dozen examples of articles on in vivo transfection with magnetic particles. It should be noted that the results on in vivo magnetofection are contradictory. In Refs. [84,100], authors mentioned that the transfection rates were comparable, as it was demonstrated with viral vectors. In Ref. [97], authors revealed that the process worked even less efficiently than when using traditional PEI/pDNA. The author in Ref. [98] did not observe differences between experiments with or without a magnetic field. More than half of the analyzed reports presented ambiguous descriptive results with conclusions at a qualitative level, which, of course, is not enough to understand the effectiveness of the method. 

It should be noted that there are a number of reports where a significant tumor growth delay is shown when using a magnetic field [30,89,90,95,96] (up to 50%, or up to 10 days depending on measuring method), numerical data on luciferase activity [91,94], or numerical data on significant (≥50%) silencing efficiencies [46,96]. According to our opinion, the main attention of researchers involved in the delivery of genes in vivo is either focused on other methods, on trying to modify the existing vectors based on lipoplexes, or to simplify the whole system. For example, the results presented in [89] clearly demonstrate that gene electrotransfer works more efficiently than magnetofection. It is believed that local injection is already a good way to avoid dose diffusion throughout the body in the case of a solid tumor, so the magnetic field can, in principle, not be used [98]; alternatively, for example, hyperthermia itself is now more effective than the gene therapy [70], so there is not much point in the gene therapy. There are attempts to modify existing working lipoplexes for the so-called selective organ targeting [140]. The idea is that the adjustment of internal charge of the lipoplex could mediate the tissue-specific delivery. In the literature, there are increasingly more papers on the topic of the initial transfecting of cells in vitro and their subsequent introduction into the body, into the tumor [141,142,143,144,145]. In vitro transfection is, in any case, easier to perform as there are no such negative effects as (1) undesired interactions with blood components and (2) the rapid elimination from the circulation by the reticuloendothelial system (RES). These two facts also indicate that, in the case of the systemic injection of a magnetic vector carrying DNA, the method will work even worse, as it will have to face even more difficulties. Therefore, in this review, we collected only data on magnetofection examples, which were carried out using only local injection and did not touch the systemic injection of magnetic vectors. 

It is worth noting here that the investigation of factors that affect the blood circulation of nanoparticles (particle size, zeta-potential, coating, etc.) is a critical task for the successful application of in vivo magnetofection. In this sense, our laboratory has achieved a certain success: in addition to a comprehensive study of various factors affecting the circulation time of nanoagents in the bloodstream [146], we were able to increase the circulation half-life of a range of short-circulating and long-circulating nanoparticle formulations by up to 32-fold via a new “MPS-cytoblockade” technology (via partial blocking of the mononuclear phagocyte system with self-erythrocytes sensitized with administered anti-RBC antibodies) [3]. We also studied carefully the effect of different factors on the efficiency of a macrophage blockade in vivo induced by solid nanomaterials [147], and we demonstrated that RBC-hitchhiking can boost the delivery of nontargeted particles to the lungs up to the record of 120-fold [148]. As for other aspects of in vivo magnetofection, there are several different strategies to improve existing nonviral vector systems, including the search for new hybrid magnetic materials [149], the search for or synthesis of biodegradable polymers with reduced toxicity, the addition of active targeting to target cells [150,151] to improve selectivity, and additional transport domains for efficient and targeted delivery. In all these fields, there is room for development. 

With regard to the purely physical properties of the materials used to create magnetic vectors, it must be said that the enhanced magnetic response of magnetic nanoparticles is desired, which correlates with the saturation magnetization value (Ms). It was shown that a higher Ms contributes to an enhanced gene magnetofection efficiency [91]. We also believe that the role of the magnetic field gradient, magnetic gradient forces, and spatial magnetic field distributions in focusing (targeting and/or retaining) magnetic particles and nucleic acids is significant. It is known that the magnetic gradient force is proportional to the product of the field gradient and magnetic field induction, and it is directed toward the magnetic field area with the largest gradient of the magnetic induction [152,153]; namely, the magnetic force is responsible for the precession in magnetic targeting (focusing) of complexes based on magnetic particles and nucleic acids. Thus, the magnetofection efficiency can be improved by using nonuniform magnetic fields with highly localized gradient areas [154]. Another aspect, which one should pay attention to, is magnetic dipole interactions between the nanoparticles. Due to these interactions, dense nanoparticle assemblies may form. They can significantly affect the sensing of the external magnetic field by magnetic nanoparticles. Other parameters such as the quality of nanoparticles, role of magnetic anisotropy, and the role of the size of magnetic nanoparticles are also important. However, we should state that reviewing the role of each of these physicochemical variables in focusing (targeting and/or retaining) magnetic particles and nucleic acids is a complex topic that needs a separate, thorough, and comprehensive review, and it is still beyond the scope of this work. However, we should take all these parameters into account when designing in vivo magnetofection experiments.

Therefore, the development of safe, stable, effective, and tumor-specific nanoparticles remains unfulfilled; however, according to our opinion, it is an achievable goal for the future successful clinical applications of the gene therapy based on magnetic nanoparticles.

## Figures and Tables

**Table 1:** Target (tissue/organ), nucleic acid type, magnetic nanoparticle composition, cell lines tested in vitro, and available characteristics of magnetic field summarized from the literature data.

**Target (Tissue/Organ)**	**Animals**	**Nucleic Acid Type ^a^**	**Cell Lines Tested in Vitro ^b^**	**Magnetic Nanoparticle Composition ^c^**	**Comparing Results with and Without Magnetic Field**	**Ref.**	**Magnetic Field (Gradient), T (T/m)**
hippocampus	mouse	pDNA	293T, PHNC	MNP + PLGA + PEI + PEG	+	[83]	-
cerebral cortex	rat	pDNA	-	NeuroMag (OzBiosciences)	+	[84]	-
circular smooth of perianal region	rat	siRNA miRNA	-	PolyMag (Oz Biosciences)	-	[85]	-
skull	mouse	pDNA	ASCs	MNP + PEI + PBAE	+	[86]	1.2
brain	mouse	pDNA	CHO-K1	γ-Fe_2_O_3_ + CaP	-	[87,88]	0.24
subcutaneous tumor	mouse	pDNA	B16F1, B16F10, 2H-11	SPIONs + PAA + PEI	+	[89,90]	0.4 (38)
subcutaneoustumor	mouse	pDNA	B16F10, HepG2	Fe_3_O_4_@SiO_2_-COOH + PEI	+	[91]	-
scapular region/thoracic wall (subcutaneous tumor)	cat	pDNA	-	transMAG^PEI^ (Chemicell)	-	[92,93]	-
Lungs/heart	mouse	pDNA	NIH3T3, HEK293, COS7	MNBs (MiltenyiBiotec)-PEI	+	[94]	-
dorsalflank (subcutaneous tumor)	mouse	pDNA	SACC-83	Fe_3_O_4_ -PEI	+	[95]	-
rightflank (subcutaneous tumor)	mouse	pDNA	B16F1, SK-MEL-28, MeT-5A, L929	SPIONs-PAA-PEI	+	[96]	-
ileum (rat), ear veins (pig)	rat, pig	pDNA	K562, PBL	transMAG^PEI^ (Chemicell)	+	[46]	-
spinal cord	rat	pDNA	U87, H4, T98G, NT2 (Ntera-2/D1), U251	PolyMag(Chemicell)-Tat	-	[48]	1.21
nasal epithelium	mouse	pDNA	C127	transMAG^PEI^ + GL67	+	[97]	1.08–1.15
rightflank (subcutaneous tumor)	mouse	pDNA	LoVo	MGN (GodMag)	+	[98]	0.5
radial bone defect	rabbit	pDNA	HUVEC-1, MG-63	Fe_3_O_4_ + Chitosan	+	[99]	0.2/0.8
skin	rat	pDNA	-	fluidMAG-Tween60 (Chemicell) + magnetobubbles	-	[100]	-
thigh muscle	mouse	pDNA	BHK-21, Hela, CHO	BMPs + PEI	+	[101]	0.5
tibialisanterior muscle	mouse/rabbit	pDNA	COS-7	transMAG^PEI^ (Chemicell)	+	[102]	0.4
striatum	rat	AntisenseODN	-	NeuroMag (OzBiosciences)	-	[103]	-
subcutaneoustumor/lungs	mouse	pDNA	B16F10, LLC1	BMPs	+	[104]	-

^a^ pDNA = plasmid DNA, siRNA = small interfering RNA, miRNA = microRNA, Antisense ODN = antisense oligonucleotide, ^b^ 293T = human cell line, derived from the HEK293 cell line, which expresses a mutant version of the SV40 large T antigen; PHNC = primary hippocampal neuron culture from neonatal rats (P0, SD); ASCs = human adipose-derived stromal cells; CHO-K1 = sub clone of the original Chinese hamster ovary (CHO) cell line; B16F1 and B16F10 = murine melanoma cell lines; 2H-11 = murine endothelial cell line; HepG2 = human liver cancer cell line; NIH3T3 = mouse embryonic fibroblast cells; HEK293 = human embryonic kidney cells; COS7 = monkey SV40 transformed kidney fibroblast cells; SACC-83 = human salivary gland adenoid cystic carcinoma cell line; SK-MEL-28 = human melanoma cell line (ATCC HTB-72); MeT-5A = human mesothelial cells transfected with pRSV-T 5A; L929 = normal fibroblast cell line from subcutaneous connective tissue of mouse; K562 = first human immortalized myelogenous leukemia cell line; PBL = peripheral blood lymphocyte; U87 = human primary glioblastoma cell line; H4 = hypertriploid human cell line having the modal chromosome number of 73 occurring in 26% of cells; T98G = glioblastoma cell line; NT2(Ntera-2/D1) = lung malignant pluripotent embryonal carcinoma cell line; U251 = glioblastoma cell line; C127 = murine mammary tumor cell line; LoVo = human colon cancer cell line; HUVEC-1 = human umbilical vein endothelial cells; MG-63 = human osteogenic sarcoma cells; BHK-21 = mouse renal cell line; Hela = human cervical adenocarcinoma cell line; LLC1 = cell line established from the lung of a C57BL mouse bearing a tumor resulting from an implantation of primary Lewis lung carcinoma; ^c^ MNP = magnetic nanoparticle; PLGA = poly(lactic-co-glycolic acid); PEI = polyethylenimine; PEG = polyethylene glycol; NeuroMag (OzBiosciences) = commercial magnetofection reagent for neurons; PolyMag (Oz Biosciences) = commercial magnetofection reagent; PBAE = poly-ß-amino ester; SPIONs = superparamagnetic iron oxide nanoparticles; CaP = calcium phosphate; PAA = polyacrylic acid; combiMAG = commercial magnetofection reagent; transMAGPEI (Chemicell) = commercial polyethylenimine (PEI)—coated iron oxide nanoparticles; MNB = magnetic nanobeads (MiltenyiBiotec, Auburn, CA, USA); PolyMag(Chemicell) = commercial iron oxide nanoparticles; Tat = cell-penetrating peptide; GL67 = cationic lipid Genzyme lipid (GL) 67; MGN (GodMag) = commercial magnetic gold nanoparticle; fluidMAG-Tween60 (Chemicell) = commercial iron oxide nanoparticles; BMPs = bacterial magnetic particles.

## Data Availability

Not applicable.
